# Combinational Treatment of Bioscaffolds and Extracellular Vesicles in Spinal Cord Injury

**DOI:** 10.3389/fnmol.2019.00081

**Published:** 2019-04-12

**Authors:** Xizhi Wang, Benson O. A. Botchway, Yong Zhang, Jiaying Yuan, Xuehong Liu

**Affiliations:** ^1^Department of Histology and Embryology, Medical College, Shaoxing University, Zhejiang, China; ^2^Institute of Neuroscience, Zhejiang University School of Medicine, Hangzhou, China

**Keywords:** spinal cord injury, stem cell therapy, neuronal damage, functional and axonal regeneration, bioscaffolds, extracellular vesicles

## Abstract

Spinal cord injury (SCI) can result in an irreversible disability due to loss of sensorimotor function below the lesion. Presently, clinical treatments for SCI mainly include surgery, drugs and postoperative rehabilitation. The prospective roles of bioscaffolds and exosomes in several neurological diseases have been reported. Bioscaffolds can reconnect lesion gaps as well as transport cells and bioactive factors, which in turn can improve axonal and functional regeneration. Herein, we explicate the respective roles of bioscaffolds and exosomes in SCI, and elucidate on the usage of combinational therapy involving bioscaffolds and extracellular vesicles (EVs) in improving SCI.

## Introduction

Spinal cord injury (SCI) is a severe neurological trauma with high morbidity and mortality (Ni et al., 2015). The pathophysiological mechanism of SCI is still unclear. SCI pathological processes are divided into primary and secondary injury processes (Ozturk et al., 2018). We have extensively elucidated these SCI pathological processes in our previous reports (Liu et al., 2018; Xu L. et al., 2018; Zhou et al., 2018). In the event of SCI, the routine management will involve surgical, medicinal and rehabilitation therapies (Sandrow-Feinberg and Houlé, 2015; Frank and Roynard, 2018). Owing to the ineffectiveness of these therapies, the search for effective treatment strategies for SCI is of prime importance, most especially to clinicians and patients. There have been suggestions that the potential usage of bioscaffold and stem cell therapies could improve spontaneous functional recovery in SCI. Extracellular vesicles (EVs), a form of endogenous nanovesicles, have been and are still being studied extensively in some neurological disorders (Chopp and Zhang, 2015; Ojha et al., 2017; Osier et al., 2018). In this report, we delve into some of the studies that have been conducted pertaining to the contributory roles of bioscaffolds and EVs in SCI and present our perspective on the usage of combined bioscaffold-EVs in SCI.

## The Effects of Bioscaffolds in SCI Treatment

Regeneration is hampered after SCI. This is because the microenvironment created following SCI is not conducive for cell migration and axonal growth. Axonal regulation is a vital part in nerve repair. Bioscaffolds can ameliorate the spinal cord microenvironment and direct cell behaviors such as migration, proliferation and differentiation (Caicco et al., 2013). Current research studies are focused on developing scaffolds that could steer axonal regeneration and reduce scar tissue formation. The combination of biomaterials with stem cells did improve SCI functional recovery (Khaing et al., 2014; Zweckberger et al., 2016). Biomaterial scaffolds have the ability to create a substrate where cell growth could be engineered in a highly controlled fashion (Mackenzie and Rademakers, 2008; Hakim et al., 2015). The combination of growth factors and biomaterial scaffolds demonstrated effective SCI repair by decreasing lesion cavity, promoted vascular formation and increased neural cell attachment and axonal outgrowth (Grulova et al., 2015). Spinal cord injured neurons have limited growth potential, and an adverse microenvironment could potentiate the differentiation of neural stem cells (NSCs) into astrocytes and oligodendrocytes rather than neurons (Fan et al., 2017; Piltti et al., 2017). Thus, in the event of SCI, a favorable microenvironment that has the capacity to promote NSCs differentiation and improve neurological functions will be paramount. Scaffolds possess superior biocompatibility and low immunogenicity, thus being able to establish a favorable microenvironment for SCI (Altinova et al., 2014; Takashima et al., 2015). Although the application of an ideal bioscaffold for SCI treatment in the clinical setting is presently of great interest, its potentiality in instigating inflammation is a drawback. Scaffolds can potentially induce immune responses in patients (Theodore et al., 2016; Zhao et al., 2017).

**Figure 1:**
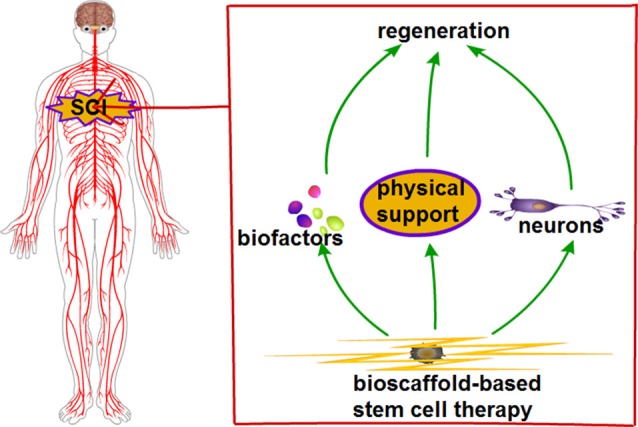
Ideal bioscaffold-based stem cell therapy for spinal cord injury (SCI).

The capability of scaffolds in maintaining the normal state in cell differentiation processes is required for SCI recovery (Kadoya et al., 2016). Scaffold features, which include non-toxicity, non-carcinogenic, biocompatible and biodegradable are imperative for SCI therapy (Novikova et al., 2018). In the event of SCI, stem cell transplantations could potentially replace lost tissue components, contribute to remyelination of damaged axons and secrete growth factors (Karamouzian et al., 2012; Shin et al., 2018). Transplanted cells include embryonic/neuronal stem cells, mesenchymal stem cells (MSCs), Schwann cells and olfactory ensheathing cells (Vismara et al., 2017; Yang et al., 2017). Scaffold and transplantation of selective stem cells might remedy the issue of regeneration in SCI. Bioscaffolds could combine with stem cells; with the combination providing physical support for the lesion gap and steering cell migration, proliferation and differentiation. In favorable conditions, stem cells could differentiate into neurons and secrete growth factors. Bioscaffold-based stem cell therapy could provide a favorable microenvironment. Taken together, the features of combined bioscaffold-stem cell could improve SCI (Figure 1).

## Prospective Role of EVs in SCI Treatment

### EVs

EVs are membrane vesicles that are released from a variety of cell types into the extracellular space (van der Pol et al., 2012). EVs mainly consist of exosomes (diameter: 30–100 nm), microvesicles (diameter: 100–1,000 nm) and apoptotic bodies (diameter: 1,000–5,000 nm; van der Pol et al., 2012; Iessi et al., 2017; Caruso Bavisotto et al., 2019). EVs have gained much attention in recent years (Verma et al., 2015). EVs were first discovered in sheep reticulocytes in 1983, with its name, “exosome,” coined in 1987 (Pan and Johnstone, 1983; Johnstone et al., 1987). EVs are small vesicles that contain nucleic acids such as DNA, messenger RNA (mRNA), long non-coding RNA (lncRNA), microRNA (miRNA), proteins and lipids (Hong et al., 2017; Mo et al., 2018; Torralba et al., 2018). EVs originate from the inward budding of cells called multivesicular bodies (MVBs). Undergoing a maturation process, intraluminal vesicles (pre-exosomes) that have accumulated in the MVBs blend with the plasma membrane, subsequently releasing EVs (Caruso Bavisotto et al., 2019). EVs’ life journey includes endosome biogenesis, transport, release and re-uptake by endocytosis (Wang et al., 2017).

EVs can be extricated by several methods including ultracentrifugation, filtration centrifugation, density gradient centrifugation and immunomagnetic separation (Schageman et al., 2013; Brownlee et al., 2014; Muller et al., 2014). A number of cells can secrete EVs in both normal and pathological conditions. EVs are naturally present in body fluids, including blood, saliva, urine, cerebrospinal fluid (CSF) and breast milk (Prada and Meldolesi, 2016). The numerous sources of EVs show their potentiality of being employed in the clinical setting, as they are readily available in the event that they are needed. In other words, EVs could be used extensively if they are effective and easy to obtain. That said, EVs derived from human plasma could potentially be unsafe. For example, EVs delivering pathological prions could affect normal cells and EVs carrying miR-29b could impede the neuroprotective function in HIV patients (Hu et al., 2012; Berrone et al., 2015; Caruso Bavisotto et al., 2019). Cellular prion protein (PrPC) from plasma-derived EVs might contribute to the pathogenesis and propagation of prion diseases such as transmissible spongiform encephalopathies (TSEs), a type of neurodegenerative disease (Berrone et al., 2015; Properzi et al., 2015b). The study by Nonaka et al. (2013) reported TAR DNA-binding protein 43 (TDP-43) aggregates to be potentially conveyed from one cell to the other, to some extent, *via* EVs. Aggregates of TDP-43 are implicated in both frontotemporal dementia and amyotrophic lateral sclerosis (ALS) with all these studies put across, choosing the safest source of exosomes is paramount. In this regard, we refer to the article by Campanella et al. (2019). One of the suggested choices out across by the authors was the employment of EVs that had could be extracted from the same patient so as to avoid the issue of immunogenicity-associated complications. These EVs, of course, would have to be subjected to therapies and tinkering. The only hindrance to this choice, however, will be the lengthened time that it will take for the therapy to be initiated following injury. The secretion of EVs by host cells to recipient cells could regulate the biological activities of the recipient cells through the substances that the EVs carry (De Toro et al., 2015). The molecular mechanisms involved in the secretion, uptake, transmission of signals and corresponding functions between cells are still unclear.

### Potential Applications of EVs

EVs are involved in intercellular communication and play important roles in the regulation of stem cell maintenance, tissue repair and immunosurveillance (Katsman et al., 2012; Robbins and Morelli, 2014; Benito-Martin et al., 2015; Rani et al., 2015). EVs can transport molecules and modulate biological functions within recipient cells (Montecalvo et al., 2012; Logozzi et al., 2019). The multiple functions of EVs and their superiority such as small size have raised the possibilities for their development and usage as therapeutic, diagnostic and screening purposes for some diseases. For instance, miRNA-derived EVs could be used as non-invasive biomarkers to screen and diagnose lung cancer (Cazzoli et al., 2013). As a messenger of information exchange between cells, EVs could potentially transmit genetic information and proteins through the following manner: (1) EVs membrane can fuse with target cell membrane and directly release its RNAs or proteins directly; (2) EVs membrane protein can bind to target cell membrane protein and activate a series of signaling pathways; and (3) EVs membrane proteins can be cleaved by proteases in the extracellular matrix, with the cleaved fragments acting as ligands to bind to receptors on the target cell membrane and cause a cascade reaction (Wahlgren et al., 2012; Mulcahy et al., 2014; Yoon et al., 2014). EVs could be internalized by recipient cells through mechanisms such as endocytosis including clathrin-mediated endocytosis, phagocytosis, macropinocytosis and plasma or endosomal membrane fusion. Endocytosis is an energy-dependent process and is indicative of endocytic pathways (Mulcahy et al., 2014). The size, contents and membrane composition of EVs are heterogeneous and depend on the cellular source, state and environmental conditions. The function of exosomes also depends on the type of cells where they are derived, which could be utilized in many applications such as immune response, antigen presentation, cell migration and differentiation, and tumor invasion (Frydrychowicz et al., 2015; Pusic et al., 2016). Tumor-derived EVs were involved in the exchange of genetic information between tumor cells and normal cells, which was impetus to tumor invasion or inhibition (Zech et al., 2012). The study by Cossetti et al. (2014) reported the possible key role of EVs in serving as carriers in the soma-to-germline transmission of nucleic acids (specifically RNA). EVs are involved in tumor metastasis and chemotherapeutic drug resistance, which might be the cause of tumor refractoriness (Federici et al., 2014; Xiao et al., 2014; Kreger et al., 2016; Lugini et al., 2016). Exactly, in turn, EVs-mediated treatments in tumors attracted the attention of many researchers (Jang et al., 2013; Pascucci et al., 2014; Saari et al., 2015; Kim M. S. et al., 2018). The use of EVs as a delivery mechanism of chemotherapeutics such as cisplatin, doxorubicin and highly cytotoxic drugs such as acridine orange increases the therapeutic index of a tumor (Toffoli et al., 2015; Hadla et al., 2016; Agrawal et al., 2017; Iessi et al., 2017). While the vast majority of these studies showed a promising drug delivery system of EVs in cancer, EVs could be applied in other conditions such as SCI owing to its delivery efficacy, low immunogenicity and high biocompatibility.

EVs act on the innate immune system as paracrine messengers and have been described as pro-inflammatory mediators in many chronic inflammatory diseases, such as rheumatoid arthritis and atherosclerosis (Boilard et al., 2010; Holder et al., 2012; Hoyer et al., 2012). They also exert immunomodulatory properties against both infectious agents and tumors, and alleviate immune abnormalities such as graft-vs.-host disease (Kordelas et al., 2014). On the basis of these features, EVs could play a contributory role in both the diagnosis and treatment of immune-related or inflammatory diseases. Recently, researches on EVs in many fields are in full swing (Bei et al., 2017; Fang et al., 2018; Huang et al., 2018; Li H. et al., 2018; Nojehdehi et al., 2018). Also, EVs could be used for cell co-culturing *in vitro* or *in vivo* injection with biological activity. Another key feature of EVs is their ability to cross the blood-brain barrier (BBB; Zhuang et al., 2011; Chen et al., 2016). EVs in both circulation and the CSF could make these vesicles unfold long-distance communication and transport bioactive molecules to selected targets. Circulating EVs could reveal the status of the tissue origin and provide an accurate means for minimally invasive diagnosis of neurological diseases. EVs have been applied as drug delivery vehicles in Parkinson’s disease (Haney et al., 2015). Experiments in rats showed MSCs-derived EVs attenuated inflammation and demyelination of the central nervous system (CNS; Li Z. et al., 2018).

### Contribution of EVs in SCI Treatment (Table 1)

Results from published EVs studies have been encouraging, which in turn may increase their potential of being applied in SCI (Xin et al., 2013; Properzi et al., 2015a; Zhang et al., 2015; Kim et al., 2016; Liu et al., 2019; Ren et al., 2019). The innate immune response plays a role in neuroinflammation following CNS injury *via* activation of inflammasomes (Haneklaus et al., 2013; de Rivero Vaccari et al., 2016). The expressions of nucleotide-binding-and-oligomerization domain (NOD)-like receptor protein-1 (NLRP-1) inflammasome, apoptosis-associated speck-like protein containing a caspase recruitment domain (ASC) and caspase-1 are significantly elevated in spinal cord neurons following trauma. NLRP1 inflammasome proteins are present in EVs derived from CSF after SCI. EVs-mediated short-interfering RNA (siRNA) delivery inhibited the inflammatory process following SCI (de Rivero Vaccari et al., 2016). Owing to the lack of effective treatment for the primary phase of SCI, the inhibition of the secondary phase of SCI by effective measures could potentially curtail SCI-associated disabilities. One of these effective measures, we believe, lies in the application of EVs due to its numerous beneficial factors. EVs released from mesenchymal stromal cells attenuated apoptosis, inflammation and promoted angiogenesis following SCI (Huang et al., 2017). Human umbilical cord MSCs-derived EVs promoted functional recovery in SCI mice by curtailing inflammation (Sun et al., 2018). EVs derived from miR-133b-modified MSC promoted recovery after SCI (Li D. et al., 2018). MSC-derived EVs reduced SCI-induced A1 astrocytes and exerted anti-inflammatory and neuroprotective effects following SCI (Wang et al., 2018). The EVs isolated from differentiated PC12 cells and MSCs exerted a protective role in SCI treatment by inhibiting the expression of phosphatase and tensin homolog (PTEN; Xu G. et al., 2018). All these published studies evince the therapeutic role of exosomes in SCI. Although the specific mechanisms pertaining to the therapeutic effects of EVs in SCI have not been clearly defined, there is the possibility that the mechanisms might involve an inhibitory effect on neuronal cell apoptosis and inhibition of inflammatory responses in a series of signaling pathways: (1) Active Wnt proteins are secreted on EVs; (2) EVs could transfer epidermal growth factor receptor (EGFR) to endothelial cells, and subsequently activate both MAPK and Akt pathways; (3) Exosomal miR-9 could stimulate angiogenesis by activating JAK-STAT signal pathway; (4) EVs from activated CD8+ T-cell could activate ERK and NF-κB pathways; and (5) nanovesicles could activate the JNK and c-Jun signaling cascades in MSCs (Al-Nedawi et al., 2009; Cai et al., 2012; Zhuang et al., 2012; Kim H. Y. et al., 2018). In view of the fact that the precise pathophysiology of EVs in SCI presently remains unclear, these related signaling pathways warrant further studies as they could potentially shed light on EVs’ pathophysiology.

**Table 1:** Summary of applications of exosomes.

Applications	Sample source	Conclusions	References
Lung cancer	Human	Exosomes may serve as minimally invasive diagnostic applications.	Cazzoli et al. (2013)
Cancer-directed immune response	Rat	Exosomes may distinctly affect the immune system.	Zech et al. (2012)
Preeclampsia	Human	Microvesicles can modulate immune cell responsiveness at different times of pregnancy and in preeclampsia.	Holder et al. (2012)
Graft-versus-host Disease (GvHD)	Human	Mesenchymal stem cells-exosome therapy improved clinical GvHD symptoms significantly.	Kordelas et al. (2014)
Type-1 diabetes mellitus (T1DM)	Mice	Exosomes exert ameliorative effects on autoimmune T1DM.	Nojehdehi et al. (2018)
Colorectal cancer	Human	Exosomes derived from hypoxic colorectal cancer enhance prometastatic behaviors and may provide new targets for colorectal cancer treatment.	Huang et al. (2018)
Cardiac ischemia-reperfusion injury	Mice	Exercise-derived extracellular vesicles might serve as a potent therapy for myocardial injury in the future.	Bei et al. (2017)
Hepatocellular carcinoma	Human	Exosomal transfer of siGRP78 can suppress Sorafenib resistance in hepatocellular carcinoma.	Li H. et al. (2018)
Steroid-induced femoral head necrosis (SFHN)	Rat	Exosomes affect SFHN osteogenesis and may develop a novel therapeutic agent for SFHN.	Fang et al. (2018)
Parkinson’s disease	Mouse	Exosomes loaded with catalase produce a neuroprotective effect.	Haney et al. (2015)
Autoimmune encephalomyelitis	Rat	Exosomes may be a promising cell-free therapy for multiple sclerosis.	Li Z. et al. (2018)
Central nervous system (CNS) trauma	Human	Exosomes can deliver siRNA into the CNS to decrease inflammasome activation.	de Rivero Vaccari et al. (2016)
Traumatic brain injury (TBI)	Rat	Exosomes effectively improve functional recovery in rats after TBI.	Zhang et al. (2015)
Stroke	Rat	Exosomes can be employed for stroke treatment.	Xin et al. (2013)

**Figure 2:**
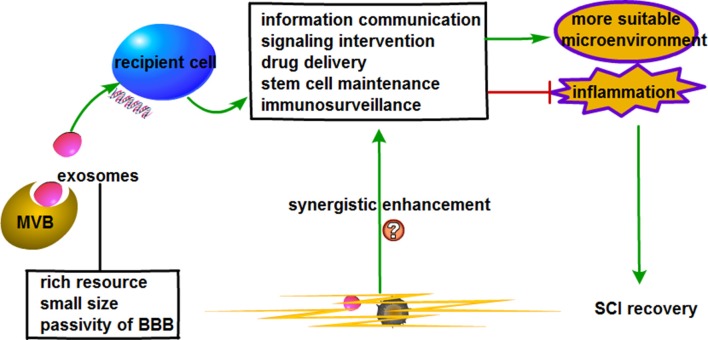
Prospective role of extracellular vesicles (EVs) combined with bioscaffold in stem cell therapy for SCI.

EVs are characterized by their ability to transfer proteins and genetic information to instruct intercellular communication. Through the employment of EVs, we are of the view that the alteration of detrimental messages produced in damaged tissues could potentially be instrumental in SCI treatment. SCI could be improved by loading certain substances that have anti-inflammatory effects. Since the instigation of signaling pathways by inflammatory factors is one of SCI pathogenesis, EVs could be employed to inhibit the inflammatory process following injury. For example, a specific inhibitor of certain signaling pathways could be combined with EVs and injected into the injured spinal cord. Also, the encapsulation and transportation of proteins, RNAs and drugs might provide novel insights for the treatment of diseases such as SCI. RNAs and proteins packaged within EVs are stable, thus, increasing their potential application in clinical therapies. In two separate animal studies, mesenchymal stromal cells-derived EVs promoted neurovascular plasticity and functional recovery in stroke and traumatic brain injury (Xin et al., 2013; Zhang et al., 2015). Since EVs have the potential to exert beneficial therapeutic effects in these neurological diseases, we believe it might exert similar effects in SCI. That said, a thorough pre-clinical and clinical studies are still needed to further evidence its suitability in SCI therapy (Figure 2).

## Combination of EVs and Biological Scaffolds

We do believe a substantial improvement in SCI repair could lie in the usage of combinational treatment methods. Though several combinations have been evidenced to be significantly better than single treatments, the complete recovery following severe SCI has not been found (Tabesh et al., 2009). One of the promising SCI therapies would be to incorporate biodegradable polymer grafts with other therapeutic strategies. The study by Huleihel et al. (2016) showed that vesicles (from “exomere”-sized particles to “microvesicles” of 1,000 nm) are associated with scaffolds placed in different organ sites and that those vesicles have different miRNA signatures, implying they might come from tissue-specific cellular sources. These “inherent” vesicles exhibited certain *ex-vivo* effects on neurite-like outgrowth from neuroblastoma cells and promoted an M2-type macrophage phenotype. Thus, the vesicles already present might influence the surrounding cellular composition. It would, therefore, be safe and logical to hypothesize that activated astrocytes and microglia in a SCI setting could release pro-inflammatory vesicles that could reside for potentially long periods of time on these scaffolds, and possibly aid in the maintenance of the inflammatory environment. Implantation of an ideal scaffold could potentially inhibit glial scar formation and guide the orientated axonal growth along the scaffold. This, in turn, could reconnect neuronal relays between nascent and host neurons and facilitate recovery of neurological function after SCI. Additionally, an ideally modified scaffold integrating bioactive factors might potentially exhibit neuroprotective effects through its dispensation of physical support to bridge the lesion gap following spinal cord transection or resection, as well as providing guidance cues in nerve fiber regrowth and functional regeneration of neural stem cells. Owing to the microenvironment affecting spinal cord repair after injury, the provision of a suitable microenvironment as well as the inhibition of adverse environments during SCI repair is paramount. Scaffold implantation could potentially induce immune responses or inflammation. This, of course, would not be conducive to SCI repair and would limit its clinical therapeutic employment. In view of this, we hypothesize that EVs combined with biological scaffold might hold promise for spinal cord regeneration across the injured site with fewer side effects. Also, the delivery of selective cargo in EVs that attenuates adverse effects while exerting their therapeutic effects would improve SCI. Stem cells have the potential of serving as effective therapeutics for severe SCI provided that: (1) the activation of biological factors is strictly controlled; (2) the differentiation profiles of stem cells are properly regulated; and (3) the microenvironment is suitable. One risk of stem cells application is their potential of resulting in tumorigenesis (Rodriguez et al., 2012). MSCs administration might cause a stoppage in the distal blood vessels due to their relatively large cell size (Furlani et al., 2009). Thus, fully using the advantages of stem cells while avoiding disadvantages is a critical step toward applying them to SCI. Studies have attributed the main therapeutic functions of stem cells to the paracrine mechanisms, where EVs might be the most valuable therapeutic factor (Ratajczak et al., 2014). EVs have low immunogenicity, can cross the BBB and effectively deliver functional molecules such as siRNA, miRNA and drugs to target cells (Alvarez-Erviti et al., 2011; Zhuang et al., 2011; Fais et al., 2016). Through the employment of receptor-mediated endocytosis for internalization, EVs assisted in the delivery of drugs to target cells; this was irrespective of the concentration gradient (Wang et al., 2017). These characteristics support the feasibility of being used in SCI treatment. Also, the release of functional cargo in EVs from a suitable scaffold might play a significant role in cell surface interactions, cell proliferation and migration and interconnectivity. We do believe that in the event of SCI, stem cells-derived EVs could reduce the complication associated with scaffolds and improve the conveyance of nutrients to the injured site, which, in turn, could potentially enhance axonal regeneration.

MSC-derived EVs have been reported to be non-immunogenic in the autologous setting and is well tolerated in humans (Kusuzaki et al., 2017). Several studies have evinced the feasibility of MSC-derived EVs in SCI treatment (Kim H. Y. et al., 2018; Li D. et al., 2018; Sun et al., 2018; Wang et al., 2018; Xu G. et al., 2018; Liu et al., 2019). In the light of these, MSC-derived EVs could potentially be employed in SCI therapy. With multifarious advantages such as diverse sources, small size and ability to cross the BBB, the combination of EVs with bioscaffold might improve SCI recovery. Just as stem cells can combine with scaffold, EVs could also combine with scaffolds in a similar manner as evidenced in several studies (Gao et al., 2014; Gurruchaga et al., 2017; DeBrot and Yao, 2018). Briefly, through a series of techniques such as cell culture, stimulation and EVs isolation and identification, EVs could be blotted onto scaffold under sterile conditions and left still for hours for the EVs to be completely absorbed to finalize the combination of EVs and scaffolds (Zhang et al., 2016, 2017; Wei et al., 2019). EVs derived from human-induced pluripotent stem cell-derived MSC combined with tricalcium phosphate (β-TCP) effectively promoted bone repair and regeneration in a rat model of calvarial bone defects (Zhang et al., 2016). This study established a strong possibility and regulation for the practical study of biological scaffolds combined with EVs. On the basis of EVs features such as information communication, signaling intervention, drug delivery, stem cell sustenance and immunosurveillance, the employment of bioscaffold-based EVs therapy could potentially improve SCI.

## Conclusion and Future Perspective

Axonal regeneration of CNS in their native environment is intricate due to inhibitory functions in their extracellular environment. This, in turn, complicates the management of neurological disorders, including SCI. Several natural and synthetic polymers have been used as either scaffolds or within scaffolds for nerve regeneration. EVs that can be modified and loaded with drugs or therapeutic agents are emerging to improve SCI therapy efficiency, and exosomal cargo is an ideal biomarker that can elucidate the complex mechanisms appertaining to SCI. The stability of EVs in peripheral circulation suggests that they can be used in SCI recovery. EVs can transfer their contents to recipient cells, and can also be combined with either scaffold or stem cells, resulting in augmented neuronal differentiation and favorable microenvironment for SCI repair. In order to expedite the usage of combinational therapy involving EVs and bioscaffolds for SCI treatment in the clinical setting, we do suggest the following:

1. Thorough elucidation of the specific type of EVs/parental cells to employ in the event of SCI.
2. Thorough investigation of the gold standard approach to isolating EVs and the tools for tracking EVs production, uptake and long-term distribution;
3. Comprehensive elucidation of the precise mechanisms underlying the application of EVs such as signaling pathways;
4. Exploration of inexpensive but excellent bioscaffolds that could effectively bind and release EVs in a rational manner (i.e., exosomes that could be released at the right time and place);
5. Extensive investigation of the long-term possible adverse reactions and response measures to these adverse reactions.

Additionally, larger clinical studies together with the suggested points are also warranted. We do intend to systematically investigate some of these suggestions in our future studies.

## Author Contributions

XL designed the study. XW, BB, YZ, JY and XL prepared the first draft of the manuscript and revised the manuscript. All authors approved the final article.

## Conflict of Interest Statement

The authors declare that the research was conducted in the absence of any commercial or financial relationships that could be construed as a potential conflict of interest.

## Abbreviations

SCI: spinal cord injury
NSCs: neural stem cells
EVs: extracellular vesicles
mRNA: messenger RNA
lncRNA: long non-coding RNA
miRNA: microRNA
MVBs: multivesicular bodies
CSF: cerebrospinal fluid
PrP^C^: cellular prion protein
TSEs: transmissible spongiform encephalopathies
BBB: the blood-brain barrier
CNS: the central nervous system
NOD: nucleotide-binding-and-oligomerization domain
NLRP-1: nucleotide-binding-and-oligomerization domain-like receptor protein-1
ASC: apoptosis-associated speck like protein containing a caspase recruitment domain
siRNA: short-interfering RNA
MSCs: mesenchymal stem cells
PTEN: phosphatase and tensin homolog
EGFR: epidermal growth factor receptor
TCP: tricalcium phosphate.

## References

[B1] Agrawal A. K. Aqil F. Jeyabalan J. Spencer W. A. Beck J. Gachuki B. W.. (2017). Milk-derived exosomes for oral delivery of paclitaxel. Nanomedicine 13, 1627–1636. 10.1016/j.nano.2017.03.001 28300659

[B2] Al-Nedawi K. Meehan B. Kerbel R. S. Allison A. C. Rak J. (2009). Endothelial expression of autocrine VEGF upon the uptake of tumor-derived microvesicles containing oncogenic EGFR. Proc. Natl. Acad. Sci. U S A 106, 3794–3799. 10.1073/pnas.0804543106 19234131 PMC2656159

[B3] Altinova H. Möllers S. Führmann T. Deumens R. Bozkurt A. Heschel I.. (2014). Functional improvement following implantation of a microstructured, type-I collagen scaffold into experimental injuries of the adult rat spinal cord. Brain Res. 1585, 37–50. 10.1016/j.brainres.2014.08.041 25193604

[B4] Alvarez-Erviti L. Seow Y. Yin H. Betts C. Lakhal S. Wood M. J. (2011). Delivery of siRNA to the mouse brain by systemic injection of targeted exosomes. Nat. Biotechnol. 29, 341–345. 10.1038/nbt.1807 21423189

[B5] Bei Y. Xu T. Lv D. Yu P. Xu J. Che L.. (2017). Exercise-induced circulating extracellular vesicles protect against cardiac ischemia-reperfusion injury. Basic Res. Cardiol. 112:38. 10.1007/s00395-017-0628-z 28534118 PMC5748384

[B6] Benito-Martin A. Di Giannatale A. Ceder S. Peinado H. (2015). The new deal: a potential role for secreted vesicles in innate immunity and tumor progression. Front. Immunol. 6:66. 10.3389/fimmu.2015.00066 25759690 PMC4338782

[B7] Berrone E. Corona C. Mazza M. Vallino Costassa E. Faro M. L. Properzi F.. (2015). Detection of cellular prion protein in exosomes derived from ovine plasma. J. Gen. Virol. 96, 3698–3702. 10.1099/jgv.0.000291 26399471 PMC4804764

[B8] Boilard E. Nigrovic P. A. Larabee K. Watts G. F. Coblyn J. S. Weinblatt M. E.. (2010). Platelets amplify inflammation in arthritis via collagen-dependent microparticle production. Science 327, 580–583. 10.1126/science.1181928 20110505 PMC2927861

[B9] Brownlee Z. Lynn K. D. Thorpe P. E. Schroit A. J. (2014). A novel "salting-out" procedure for the isolation of tumor-derived exosomes. J. Immunol. Methods 407, 120–126. 10.1016/j.jim.2014.04.003 24735771 PMC4077054

[B10] Cai Z. Yang F. Yu L. Yu Z. Jiang L. Wang Q.. (2012). Activated T cell exosomes promote tumor invasion via Fas signaling pathway. J. Immunol. 188, 5954–5961. 10.4049/jimmunol.1103466 22573809

[B11] Caicco M. J. Zahir T. Mothe A. J. Ballios B. G. Kihm A. J. Tator C. H.. (2013). Characterization of hyaluronan-methylcellulose hydrogels for cell delivery to the injured spinal cord. J. Biomed. Mater. Res. A 101, 1472–1477. 10.1002/jbm.a.34454 23129254

[B12] Campanella C. Caruso Bavisotto C. Logozzi M. Marino Gammazza A. Mizzoni D. Cappello F.. (2019). On the choice of the extracellular vesicles for therapeutic purposes. Int. J. Mol. Sci. 20:E236. 10.3390/ijms20020236 30634425 PMC6359369

[B13] Caruso Bavisotto C. Scalia F. Marino Gammazza A. Carlisi D. Bucchieri F. Conway de Macario E.. (2019). Extracellular vesicle-mediated cell-cell communication in the nervous system: focus on neurological diseases. Int. J. Mol. Sci. 20:E434. 10.3390/ijms20020434 30669512 PMC6359416

[B14] Cazzoli R. Buttitta F. Di Nicola M. Malatesta S. Marchetti A. Rom W. N.. (2013). microRNAs derived from circulating exosomes as noninvasive biomarkers for screening and diagnosing lung cancer. J. Thorac. Oncol. 8, 1156–1162. 10.1097/jto.0b013e318299ac32 23945385 PMC4123222

[B15] Chen C. C. Liu L. Ma F. Wong C. W. Guo X. E. Chacko J. V.. (2016). Elucidation of exosome migration across the blood-brain barrier model *in vitro*. Cell. Mol. Bioeng. 9, 509–529. 10.1007/s12195-016-0458-3 28392840 PMC5382965

[B16] Chopp M. Zhang Z. G. (2015). Emerging potential of exosomes and noncoding microRNAs for the treatment of neurological injury/diseases. Expert Opin. Emerg. Drugs 20, 523–526. 10.1517/14728214.2015.1061993 26135408 PMC4878696

[B17] Cossetti C. Lugini L. Astrologo L. Saggio I. Fais S. Spadafora C. (2014). Soma-to-germline transmission of RNA in mice xenografted with human tumour cells: possible transport by exosomes. PLoS One 9:e101629. 10.1371/journal.pone.0101629 24992257 PMC4081593

[B20] DeBrot A. Yao L. (2018). The combination of induced pluripotent stem cells and bioscaffolds holds promise for spinal cord regeneration. Neural. Regen. Res. 13, 1677–1684. 10.4103/1673-5374.238602 30136677 PMC6128052

[B18] de Rivero Vaccari J. P. Brand F. III. Adamczak S. Lee S. W. Perez-Barcena J. Wang M. Y.. (2016). Exosome-mediated inflammasome signaling after central nervous system injury. J. Neurochem. 1, 39–48. 10.1111/jnc.13036 25628216 PMC4516699

[B19] De Toro J. Herschlik L. Waldner C. Mongini C. (2015). Emerging roles of exosomes in normal and pathological conditions: new insights for diagnosis and therapeutic applications. Front. Immunol. 6:203. 10.3389/fimmu.2015.00203 25999947 PMC4418172

[B21] Fais S. O’Driscoll L. Borras F. E. Buzas E. Camussi G. Cappello F.. (2016). Evidence-based clinical use of nanoscale extracellular vesicles in nanomedicine. ACS Nano 10, 3886–3899. 10.1021/acsnano.5b08015 26978483

[B22] Fan C. Li X. Xiao Z. Zhao Y. Liang H. Wang B.. (2017). A modified collagen scaffold facilitates endogenous neurogenesis for acute spinal cord injury repair. Acta Biomater. 51, 304–316. 10.1016/j.actbio.2017.01.009 28069497

[B23] Fang S. Li Y. Chen P. (2018). Osteogenic effect of bone marrow mesenchymal stem cell-derived exosomes on steroid-induced osteonecrosis of the femoral head. Drug Des. Devel. Ther. 13, 45–55. 10.2147/dddt.s178698 30587927 PMC6305133

[B24] Federici C. Petrucci F. Caimi S. Cesolini A. Logozzi M. Borghi M.. (2014). Exosome release and low pH belong to a framework of resistance of human melanoma cells to cisplatin. PLoS One 9:e88193. 10.1371/journal.pone.0088193 24516610 PMC3916404

[B25] Frank L. R. Roynard P. F. P. (2018). Veterinary neurologic rehabilitation: the rationale for a comprehensive approach. Top. Companion Anim. Med. 33, 49–57. 10.1053/j.tcam.2018.04.002 30236409

[B26] Frydrychowicz M. Kolecka-Bednarczyk A. Madejczyk M. Yasar S. Dworacki G. (2015). Exosomes - structure, biogenesis and biological role in non-small-cell lung cancer. Scand. J. Immunol. 81, 2–10. 10.1111/sji.12247 25359529

[B27] Furlani D. Ugurlucan M. Ong L. Bieback K. Pittermann E. Westien I.. (2009). Is the intravascular administration of mesenchymal stem cells safe? Mesenchymal stem cells and intravital microscopy. Microvasc. Res. 77, 370–376. 10.1016/j.mvr.2009.02.001 19249320

[B28] Gao S. Zhao P. Lin C. Sun Y. Wang Y. Zhou Z.. (2014). Differentiation of human adipose-derived stem cells into neuron-like cells which are compatible with photocurable three-dimensional scaffolds. Tissue Eng. Part A 20, 1271–1284. 10.1089/ten.TEA.2012.0773 24251600 PMC3993073

[B29] Grulova I. Slovinska L. Blaško J. Devaux S. Wisztorski M. Salzet M.. (2015). Delivery of alginate scaffold releasing two trophic factors for spinal cord injury repair. Sci. Rep. 5:13702. 10.1038/srep13702 26348665 PMC4562265

[B30] Gurruchaga H. Saenz Del Burgo L. Garate A. Delgado D. Sanchez P. Orive G.. (2017). Cryopreservation of human mesenchymal stem cells in an allogeneic bioscaffold based on platelet rich plasma and synovial fluid. Sci. Rep. 7:15733. 10.1038/s41598-017-16134-6 29146943 PMC5691190

[B31] Hadla M. Palazzolo S. Corona G. Caligiuri I. Canzonieri V. Toffoli G.. (2016). Exosomes increase the therapeutic index of doxorubicin in breast and ovarian cancer mouse models. Nanomedicine 11, 2431–2441. 10.2217/nnm-2016-0154 27558906

[B32] Hakim J. S. Esmaeili Rad M. Grahn P. J. Chen B. K. Knight A. M. Schmeichel A. M.. (2015). Positively charged oligo[poly(ethylene glycol) fumarate] scaffold implantation results in a permissive lesion environment after spinal cord injury in rat. Tissue Eng. Part A 21, 2099–2114. 10.1089/ten.TEA.2015.0019 25891264 PMC4507127

[B33] Haneklaus M. O’Neill L. A. Coll R. C. (2013). Modulatory mechanisms controlling the NLRP3 inflammasome in inflammation: recent developments. Curr. Opin. Immunol. 25, 40–45. 10.1016/j.coi.2012.12.004 23305783

[B34] Haney M. J. Klyachko N. L. Zhao Y. Gupta R. Plotnikova E. G. He Z.. (2015). Exosomes as drug delivery vehicles for Parkinson’s disease therapy. J. Control. Release 207, 18–30. 10.1016/j.jconrel.2015.03.033 25836593 PMC4430381

[B35] Holder B. S. Tower C. L. Jones C. J. Aplin J. D. Abrahams V. M. (2012). Heightened pro-inflammatory effect of preeclamptic placental microvesicles on peripheral blood immune cells in humans. Biol. Reprod. 86:103. 10.1095/biolreprod.111.097014 22205696

[B36] Hong C. S. Sharma P. Yerneni S. S. Simms P. Jackson E. K. Whiteside T. L.. (2017). Circulating exosomes carrying an immunosuppressive cargo interfere with cellular immunotherapy in acute myeloid leukemia. Sci. Rep. 7:14684. 10.1038/s41598-017-14661-w 29089618 PMC5666018

[B37] Hoyer F. F. Giesen M. K. Nunes França C. Lütjohann D. Nickenig G. Werner N. (2012). Monocytic microparticles promote atherogenesis by modulating inflammatory cells in mice. J. Cell Mol. Med. 16, 2777–2788. 10.1111/j.1582-4934.2012.01595.x 22697268 PMC4118246

[B38] Hu G. Yao H. Chaudhuri A. D. Duan M. Yelamanchili S. V. Wen H.. (2012). Exosome-mediated shuttling of microRNA-29 regulates HIV Tat and morphine-mediated neuronal dysfunction. Cell Death Dis. 3:e381. 10.1038/cddis.2012.114 22932723 PMC3434655

[B40] Huang Z. Yang M. Li Y. Yang F. Feng Y. (2018). Exosomes derived from hypoxic colorectal cancer cells transfer wnt4 to normoxic cells to elicit a prometastatic phenotype. Int. J. Biol. Sci. 14, 2094–2102. 10.7150/ijbs.28288 30585272 PMC6299371

[B39] Huang J. H. Yin X. M. Xu Y. Xu C. C. Lin X. Ye F. B.. (2017). Systemic administration of exosomes released from mesenchymal stromal cells attenuates apoptosis, inflammation and promotes angiogenesis after spinal cord injury in rats. J. Neurotrauma 34, 3388–3396. 10.1089/neu.2017.5063 28665182

[B41] Huleihel L. Hussey G. S. Naranjo J. D. Zhang L. Dziki J. L. Turner N. J.. (2016). Matrix-bound nanovesicles within ECM bioscaffolds. Sci. Adv. 2:e1600502. 10.1126/sciadv.1600502 27386584 PMC4928894

[B43] Iessi E. Logozzi M. Lugini L. Azzarito T. Federici C. Spugnini E. P.. (2017). Acridine orange/exosomes increase the delivery and the effectiveness of acridine orange in human melanoma cells: a new prototype for theranostics of tumors. J. Enzyme Inhib. Med. Chem. 32, 648–657. 10.1080/14756366.2017.1292263 28262028 PMC6010124

[B44] Jang S. C. Kim O. Y. Yoon C. M. Choi D. S. Roh T. Y. Park J.. (2013). Bioinspired exosome-mimetic nanovesicles for targeted delivery of chemotherapeutics to malignant tumors. ACS Nano. 7, 7698–7710. 10.1021/nn402232g 24004438

[B45] Johnstone R. M. Adam M. Hammond J. R. Orr L. Turbide C. (1987). Vesicle formation during reticulocyte maturation. J. Biol. Chem. 262, 9412–9420. 3597417

[B46] Kadoya K. Lu P. Nguyen K. Lee-Kubli C. Kumamaru H. Yao L.. (2016). Spinal cord reconstitution with homologous neural grafts enables robust corticospinal regeneration. Nat. Med. 22, 479–487. 10.1038/nm.4066 27019328 PMC4860037

[B47] Karamouzian S. Nematollahi-Mahani S. N. Nakhaee N. Eskandary H. (2012). Clinical safety and primary efficacy of bone marrow mesenchymal cell transplantation in subacute spinal cord injured patients. Clin. Neurol. Neurosurg. 114, 935–939. 10.1016/j.clineuro.2012.02.003 22464434

[B48] Katsman D. Stackpole E. J. Domin D. R. Farber D. B. (2012). Embryonic stem cell-derived microvesicles induce gene expression changes in Müller cells of the retina. PLoS One 7:e50417. 10.1371/journal.pone.0050417 23226281 PMC3511553

[B49] Khaing Z. Z. Thomas R. C. Geissler S. A. Schmidt C. E. (2014). Advanced biomaterials for repairing the nervous system: what can hydrogels do for the brain? Mater. Today 17, 332–340. 10.1016/j.mattod.2014.05.011

[B52] Kim M. S. Haney M. J. Zhao Y. Yuan D. Deygen I. Klyachko N. L.. (2018). Engineering macrophage-derived exosomes for targeted paclitaxel delivery to pulmonary metastases: *in vitro* and *in vivo* evaluations. Nanomedicine 14, 195–204. 10.1016/j.nano.2017.09.011 28982587

[B51] Kim H. Y. Kumar H. Jo M. J. Kim J. Yoon J. K. Lee J. R.. (2018). Therapeutic efficacy-potentiated and diseased organ-targeting nanovesicles derived from mesenchymal stem cells for spinal cord injury treatment. Nano Lett. 18, 4965–4975. 10.1021/acs.nanolett.8b01816 29995418

[B50] Kim D. K. Nishida H. An S. Y. Shetty A. K. Bartosh T. J. Prockop D. J. (2016). Chromatographically isolated CD63+CD81+ extracellular vesicles from mesenchymal stromal cells rescue cognitive impairments after TBI. Proc. Natl. Acad. Sci. U S A 113, 170–175. 10.1073/pnas.1522297113 26699510 PMC4711859

[B53] Kordelas L. Rebmann V. Ludwig A. K. Radtke S. Ruesing J. Doeppner T. R.. (2014). MSC-derived exosomes: a novel tool to treat therapy-refractory graft-versus-host disease. Leukemia 28, 970–973. 10.1038/leu.2014.41 24445866

[B54] Kreger B. T. Johansen E. R. Cerione R. A. Antonyak M. A. (2016). The enrichment of survivin in exosomes from breast cancer cells treated with paclitaxel promotes cell survival and chemoresistance. Cancers 8:E111. 10.3390/cancers8120111 27941677 PMC5187509

[B55] Kusuzaki K. Matsubara T. Murata H. Logozzi M. Iessi E. Di Raimo R.. (2017). Natural extracellular nanovesicles and photodynamic molecules: is there a future for drug delivery? J. Enzyme. Inhib. Med. Chem. 32, 908–916. 10.1080/14756366.2017.1335310 28708430 PMC6010042

[B58] Li Z. Liu F. He X. Yang X. Shan F. Feng J. (2018). Exosomes derived from mesenchymal stem cells attenuate inflammation and demyelination of the central nervous system in EAE rats by regulating the polarization of microglia. Int. Immunopharmacol. 67, 268–280. 10.1016/j.intimp.2018.12.001 30572251

[B57] Li H. Yang C. Shi Y. Zhao L. (2018). Exosomes derived from siRNA against GRP78 modified bone-marrow-derived mesenchymal stem cells suppress Sorafenib resistance in hepatocellular carcinoma. J. Nanobiotechnol. 16:103. 10.1186/s12951-018-0429-z 30572882 PMC6300915

[B56] Li D. Zhang P. Yao X. Li H. Shen H. Li X.. (2018). Exosomes derived from miR-133b-modified mesenchymal stem cells promote recovery after spinal cord injury. Front. Neurosci. 12:845. 10.3389/fnins.2018.00845 30524227 PMC6262643

[B59] Liu W. Wang Y. Gong F. Rong Y. Luo Y. Tang P.. (2019). Exosomes derived from bone mesenchymal stem cells repair traumatic spinal cord injury by suppressing the activation of A1 neurotoxic reactive astrocytes. J. Neurotrauma 36, 469–484. 10.1089/neu.2018.5835 29848167

[B60] Liu X. Zhang Y. Yang Y. Lin J. Huo X. Du X.. (2018). Therapeutic effect of curcumin and methylprednisolone in the rat spinal cord injury. Anat. Rec. 301, 686–696. 10.1002/ar.23729 29150987

[B61] Logozzi M. Mizzoni D. Bocca B. Di Raimo R. Petrucci F. Caimi S.. (2019). Human primary macrophages scavenge aunps and eliminate it through exosomes. Eur. J. Pharm. Biopharm. 137, 23–36. 10.1016/j.ejpb.2019.02.014 30779978

[B62] Lugini L. Valtieri M. Federici C. Cecchetti S. Meschini S. Condello M.. (2016). Exosomes from human colorectal cancer induce a tumor-like behavior in colonic mesenchymal stromal cells. Oncotarget 7, 50086–50098. 10.18632/oncotarget.10574 27418137 PMC5226570

[B42] Mackenzie I. R. Rademakers R. (2008). The role of TDP-43 in amyotrophic lateral sclerosis and frontotemporal dementia. Curr. Opin. Neurol. 21, 693–700. 10.1097/WCO.0b013e3283168d1d 18989115 PMC2869081

[B63] Mo L. J. Song M. Huang Q. H. Guan H. Liu X. D. Xie D. F.. (2018). Exosome-packaged miR-1246 contributes to bystander DNA damage by targeting LIG4. Br. J. Cancer 119, 492–502. 10.1038/s41416-018-0192-9 30038324 PMC6134031

[B64] Montecalvo A. Larregina A. T. Shufesky W. J. Stolz D. B. Sullivan M. L. Karlsson J. M.. (2012). Mechanism of transfer of functional microRNAs between mouse dendritic cells via exosomes. Blood 119, 756–766. 10.1182/blood-2011-02-338004 22031862 PMC3265200

[B65] Mulcahy L. A. Pink R. C. Carter D. R. (2014). Routes and mechanisms of extracellular vesicle uptake. J. Extracell Vesicles. 4:3. 10.3402/jev.v3.24641 25143819 PMC4122821

[B66] Muller L. Hong C. S. Stolz D. B. Watkins S. C. Whiteside T. L. (2014). Isolation of biologically-active exosomes from human plasma. J. Immunol. Methods 411, 55–65. 10.1016/j.jim.2014.06.007 24952243 PMC4260336

[B67] Ni H. Jin W. Zhu T. Wang J. Yuan B. Jiang J.. (2015). Curcumin modulates TLR4/NF-κB inflammatory signaling pathway following traumatic spinal cord injury in rats. J. Spinal Cord. Med. 38, 199–206. 10.1179/2045772313Y.0000000179 24621048 PMC4397202

[B68] Nojehdehi S. Soudi S. Hesampour A. Rasouli S. Soleimani M. Hashemi S. M. (2018). Immunomodulatory effects of mesenchymal stem cell-derived exosomes on experimental type-1 autoimmune diabetes. J. Cell. Biochem. 119, 9433–9443. 10.1002/jcb.27260 30074271

[B69] Nonaka T. Masuda-Suzukake M. Arai T. Hasegawa Y. Akatsu H. Obi T.. (2013). Prion-like properties of pathological TDP-43 aggregates from diseased brains. Cell Rep. 4, 124–134. 10.1016/j.celrep.2013.06.007 23831027

[B70] Novikova L. N. Kolar M. K. Kingham P. J. Ullrich A. Oberhoffner S. Renardy M.. (2018). Trimethylene carbonate-caprolactone conduit with poly-p-dioxanone microfilaments to promote regeneration after spinal cord injury. Acta Biomater. 66, 177–191. 10.1016/j.actbio.2017.11.028 29174588

[B71] Ojha C. R. Lapierre J. Rodriguez M. Dever S. M. Zadeh M. A. DeMarino C.. (2017). Interplay between autophagy, exosomes and hiv-1 associated neurological disorders: new insights for diagnosis and therapeutic applications. Viruses 9:E176. 10.3390/v9070176 28684681 PMC5537668

[B72] Osier N. Motamedi V. Edwards K. Puccio A. Diaz-Arrastia R. Kenney K.. (2018). Exosomes in acquired neurological disorders: new insights into pathophysiology and treatment. Mol. Neurobiol. 55, 9280–9293. 10.1007/s12035-018-1054-4 29663285

[B73] Ozturk A. M. Sozbilen M. C. Sevgili E. Dagci T. Özyalcin H. Armagan G. (2018). Epidermal growth factor regulates apoptosis and oxidative stress in a rat model of spinal cord injury. Injury 49, 1038–1045. 10.1016/j.injury.2018.03.021 29602490

[B74] Pan B. T. Johnstone R. M. (1983). Fate of the transferrin receptor during maturation of sheep reticulocytes *in vitro*: selective externalization of the receptor. Cell 33, 967–978. 10.1016/0092-8674(83)90040-5 6307529

[B75] Pascucci L. Coccè V. Bonomi A. Ami D. Ceccarelli P. Ciusani E.. (2014). Paclitaxel is incorporated by mesenchymal stromal cells and released in exosomes that inhibit *in vitro* tumor growth: a new approach for drug delivery. J. Control. Release 192, 262–270. 10.1016/j.jconrel.2014.07.042 25084218

[B76] Piltti K. M. Funes G. M. Avakian S. N. Salibian A. A. Huang K. I. Carta K.. (2017). Increasing human neural stem cell transplantation dose alters oligodendroglial and neuronal differentiation after spinal cord injury. Stem Cell Reports 8, 1534–1548. 10.1016/j.stemcr.2017.04.009 28479305 PMC5469937

[B77] Prada I. Meldolesi J. (2016). Binding and fusion of extracellular vesicles to the plasma membrane of their cell targets. Int. J. Mol. Sci. 17:E1296. 10.3390/ijms17081296 27517914 PMC5000693

[B78] Properzi F. Ferroni E. Poleggi A. Vinci R. (2015a). The regulation of exosome function in the CNS: implications for neurodegeneration. Swiss Med. Wkly. 145:w14204. 10.4414/smw.2015.14204 26561744

[B79] Properzi F. Logozzi M. Abdel-Haq H. Federici C. Lugini L. Azzarito T.. (2015b). Detection of exosomal prions in blood by immunochemistry techniques. J. Gen. Virol. 96, 1969–1974. 10.1099/vir.0.000117 25805411

[B80] Pusic K. M. Pusic A. D. Kraig R. P. (2016). Environmental enrichment stimulates immune cell secretion of exosomes that promote cns myelination and may regulate inflammation. Cell Mol. Neurobiol. 36, 313–325. 10.1007/s10571-015-0269-4 26993508 PMC4860060

[B81] Rani S. Ryan A. E. Griffin M. D. Ritter T. (2015). Mesenchymal stem cell-derived extracellular vesicles: toward cell-free therapeutic applications. Mol. Ther. 23, 812–823. 10.1038/mt.2015.44 25868399 PMC4427881

[B82] Ratajczak M. Z. Jadczyk T. Pędziwiatr D. Wojakowski W. (2014). New advances in stem cell research: practical implications for regenerative medicine. Pol. Arch. Med. Wewn. 124, 417–426. 10.20452/pamw.2355 24956404

[B83] Ren Z. W. Zhou J. G. Xiong Z. K. Zhu F. Z. Guo X. D. (2019). Effect of exosomes derived from MiR-133b-modified ADSCs on the recovery of neurological function after SCI. Eur. Rev. Med. Pharmacol. Sci. 23, 52–60. 10.26355/eurrev_201901_16747 30657546

[B84] Robbins P. D. Morelli A. E. (2014). Regulation of immune responses by extracellular vesicles. Nat. Rev. Immunol. 14, 195–208. 10.1038/nri3622 24566916 PMC4350779

[B85] Rodriguez R. Rubio R. Menendez P. (2012). Modeling sarcomagenesis using multipotent mesenchymal stem cells. Cell Res. 22, 62–77. 10.1038/cr.2011.157 21931359 PMC3351912

[B86] Saari H. Lázaro-Ibáñez E. Viitala T. Vuorimaa-Laukkanen E. Siljander P. Yliperttula M. (2015). Microvesicle- and exosome-mediated drug delivery enhances the cytotoxicity of Paclitaxel in autologous prostate cancer cells. J. Control. Release 220, 727–737. 10.1016/j.jconrel.2015.09.031 26390807

[B87] Sandrow-Feinberg H. R. Houlé J. D. (2015). Exercise after spinal cord injury as an agent for neuroprotection, regeneration and rehabilitation. Brain Res. 1619, 12–21. 10.1016/j.brainres.2015.03.052 25866284 PMC4540698

[B88] Schageman J. Zeringer E. Li M. Barta T. Lea K. Gu J.. (2013). The complete exosome workflow solution: from isolation to characterization of RNA cargo. Biomed. Res. Int. 2013:253957. 10.1155/2013/253957 24205503 PMC3800616

[B89] Shin J. E. Jung K. Kim M. Hwang K. Lee H. Kim I. S.. (2018). Brain and spinal cord injury repair by implantation of human neural progenitor cells seeded onto polymer scaffolds. Exp. Mol. Med. 50:39. 10.1038/s12276-018-0054-9 29674624 PMC5938022

[B90] Sun G. Li G. Li D. Huang W. Zhang R. Zhang H.. (2018). hucMSC derived exosomes promote functional recovery in spinal cord injury mice via attenuating inflammation. Mater. Sci. Eng. C Mater. Biol. Appl. 89, 194–204. 10.1016/j.msec.2018.04.006 29752089

[B91] Tabesh H. Amoabediny G. Nik N. S. Heydari M. Yosefifard M. Siadat S. O.. (2009). The role of biodegradable engineered scaffolds seeded with Schwann cells for spinal cord regeneration. Neurochem. Int. 54, 73–83. 10.1016/j.neuint.2008.11.002 19084565

[B92] Takashima K. Hoshino M. Uesugi K. Yagi N. Matsuda S. Nakahira A.. (2015). X-ray phase-contrast computed tomography visualizes the microstructure and degradation profile of implanted biodegradable scaffolds after spinal cord injury. J. Synchrotron Radiat. 22, 136–142. 10.1107/s160057751402270x 25537600 PMC4294026

[B93] Theodore N. Hlubek R. Danielson J. Neff K. Vaickus L. Ulich T. R.. (2016). First human implantation of a bioresorbable polymer scaffold for acute traumatic spinal cord injury: a clinical pilot study for safety and feasibility. Neurosurgery 79, E305–E312. 10.1227/neu.0000000000001283 27309344

[B94] Toffoli G. Hadla M. Corona G. Caligiuri I. Palazzolo S. Semeraro S.. (2015). Exosomal doxorubicin reduces the cardiac toxicity of doxorubicin. Nanomedicine 10, 2963–2971. 10.2217/nnm.15.118 26420143

[B95] Torralba D. Baixauli F. Villarroya-Beltri C. Fernández-Delgado I. Latorre-Pellicer A. Acín-Pérez R.. (2018). Priming of dendritic cells by DNA-containing extracellular vesicles from activated T cells through antigen-driven contacts. Nat. Commun. 9:2658. 10.1038/s41467-018-05077-9 29985392 PMC6037695

[B96] van der Pol E. Böing A. N. Harrison P. Sturk A. Nieuwland R. (2012). Classification, functions and clinical relevance of extracellular vesicles. Pharmacol. Rev. 64, 676–705. 10.1124/pr.112.005983 22722893

[B97] Verma M. Lam T. K. Hebert E. Divi R. L. (2015). Extracellular vesicles: potential applications in cancer diagnosis, prognosis and epidemiology. BMC Clin. Pathol. 15:6. 10.1186/s12907-015-0005-5 25883534 PMC4399158

[B98] Vismara I. Papa S. Rossi F. Forloni G. Veglianese P. (2017). Current options for cell therapy in spinal cord injury. Trends Mol. Med. 23, 831–849. 10.1016/j.molmed.2017.07.005 28811172

[B99] Wahlgren J. De L. Karlson T. Brisslert M. Vaziri Sani F. Telemo E.. (2012). Plasma exosomes can deliver exogenous short interfering RNA to monocytes and lymphocytes. Nucleic Acids Res. 40:e130. 10.1093/nar/gks463 22618874 PMC3458529

[B101] Wang L. Pei S. Han L. Guo B. Li Y. Duan R.. (2018). Mesenchymal stem cell-derived exosomes reduce a1 astrocytes via downregulation of phosphorylated NFκB P65 subunit in spinal cord injury. Cell Physiol. Biochem. 50, 1535–1559. 10.1159/000494652 30376671

[B100] Wang J. Yeung B. Z. Cui M. Peer C. J. Lu Z. Figg W. D.. (2017). Exosome is a mechanism of intercellular drug transfer: application of quantitative pharmacology. J. Control. Release 268, 147–158. 10.1016/j.jconrel.2017.10.020 29054369 PMC5722714

[B102] Wei F. Li M. Crawford R. Zhou Y. Xiao Y. (2019). Exosome-integrated titanium oxide nanotubes for targeted bone regeneration. Acta Biomater. 86, 480–492. 10.1016/j.actbio.2019.01.006 30630122

[B103] Xiao X. Yu S. Li S. Wu J. Ma R. Cao H.. (2014). Exosomes: decreased sensitivity of lung cancer A549 cells to cisplatin. PLoS One 9:e89534. 10.1371/journal.pone.0089534 24586853 PMC3931805

[B104] Xin H. Li Y. Cui Y. Yang J. J. Zhang Z. G. Chopp M. (2013). Systemic administration of exosomes released from mesenchymal stromal cells promote functional recovery and neurovascular plasticity after stroke in rats. J. Cereb. Blood Flow. Metab. 33, 1711–1715. 10.1038/jcbfm.2013.152 23963371 PMC3824189

[B105] Xu G. Ao R. Zhi Z. Jia J. Yu B. (2018). miR-21 and miR-19b delivered by hMSC-derived EVs regulate the apoptosis and differentiation of neurons in patients with spinal cord injury. J. Cell. Physiol. 234, 10205–10217. 10.1002/jcp.27690 30387159

[B106] Xu L. Botchway B. O. A. Zhang S. Zhou J. Liu X. (2018). Inhibition of NF-κB signaling pathway by resveratrol improves spinal cord injury. Front. Neurosci. 12:690. 10.3389/fnins.2018.00690 30337851 PMC6180204

[B107] Yang E. Z. Zhang G. W. Xu J. G. Chen S. Wang H. Cao L. L.. (2017). Multichannel polymer scaffold seeded with activated Schwann cells and bone mesenchymal stem cells improves axonal regeneration and functional recovery after rat spinal cord injury. Acta Pharmacol. Sin. 38, 623–637. 10.1038/aps.2017.11 28392569 PMC5457698

[B108] Yoon Y. J. Kim O. Y. Gho Y. S. (2014). Extracellular vesicles as emerging intercellular communicasomes. BMB Rep. 47, 531–539. 10.5483/bmbrep.2014.47.10.164 25104400 PMC4261509

[B109] Zech D. Rana S. Büchler M. W. Zöller M. (2012). Tumor-exosomes and leukocyte activation: an ambivalent crosstalk. Cell Commun. Signal. 10:37. 10.1186/1478-811x-10-37 23190502 PMC3519567

[B111] Zhang Y. Chopp M. Meng Y. Katakowski M. Xin H. Mahmood A.. (2015). Effect of exosomes derived from multipluripotent mesenchymal stromal cells on functional recovery and neurovascular plasticity in rats after traumatic brain injury. J. Neurosurg. 122, 856–867. 10.3171/2014.11.jns14770 25594326 PMC4382456

[B112] Zhang Y. Chopp M. Zhang Z. G. Katakowski M. Xin H. Qu C.. (2017). Systemic administration of cell-free exosomes generated by human bone marrow derived mesenchymal stem cells cultured under 2D and 3D conditions improves functional recovery in rats after traumatic brain injury. Neurochem. Int. 111, 69–81. 10.1016/j.neuint.2016.08.003 27539657 PMC5311054

[B110] Zhang J. Liu X. Li H. Chen C. Hu B. Niu X.. (2016). Exosomes/tricalcium phosphate combination scaffolds can enhance bone regeneration by activating the PI3K/Akt signaling pathway. Stem Cell Res. Ther. 7:136. 10.1186/s13287-016-0391-3 27650895 PMC5028974

[B113] Zhao Y. Tang F. Xiao Z. Han G. Wang N. Yin N.. (2017). Clinical study of neuroregen scaffold combined with human mesenchymal stem cells for the repair of chronic complete spinal cord injury. Cell Transplant. 26, 891–900. 10.3727/096368917x695038 28185615 PMC5657723

[B114] Zhou J. Huo X. Botchway B. O. A. Xu L. Meng X. Zhang S.. (2018). Beneficial effects of resveratrol-mediated inhibition of the mTOR pathway in spinal cord injury. Neural. Plast. 2018:7513748. 10.1155/2018/7513748 29780409 PMC5892236

[B115] Zhuang G. Wu X. Jiang Z. Kasman I. Yao J. Guan Y.. (2012). Tumour-secreted miR-9 promotes endothelial cell migration and angiogenesis by activating the JAK-STAT pathway. EMBO J. 31, 3513–3523. 10.1038/emboj.2012.183 22773185 PMC3433782

[B116] Zhuang X. Xiang X. Grizzle W. Sun D. Zhang S. Axtell R. C.. (2011). Treatment of brain inflammatory diseases by delivering exosome encapsulated anti-inflammatory drugs from the nasal region to the brain. Mol. Ther. 19, 1769–1779. 10.1038/mt.2011.164 21915101 PMC3188748

[B117] Zweckberger K. Ahuja C. S. Liu Y. Wang J. Fehlings M. G. (2016). Self-assembling peptides optimize the post-traumatic milieu and synergistically enhance the effects of neural stem cell therapy after cervical spinal cord injury. Acta Biomater. 42, 77–89. 10.1016/j.actbio.2016.06.016 27296842

